# Compelling therapy of LVH: straight (and not-so-straight) inferences from evidence

**DOI:** 10.1186/s40885-019-0131-y

**Published:** 2019-11-15

**Authors:** Ravi Tejraj Mehta, Anil Pareek, Shruti Dharmadhikari

**Affiliations:** Ipca Laboratories Limited, Kandivli Industrial Estate, Kandivli (West), Mumbai, Maharashtra India

**Keywords:** Guidelines, Chlorthalidone, Amlodipine, Hypertension, Left ventricle hypertrophy, Heart failure, Thiazide diuretics, Calcium channel blockers, ACE-inhibitors

## Abstract

We have read with interest the Korean Society of Hypertension guidelines for the management of hypertension and congratulate the Society for an extensive review of literature while drafting the guidelines. The guidelines indicate preferring ACE-I and CCB over diuretics in patients with left ventricle hypertrophy. However, in landmark head-to-head comparison trials, the thiazide-like diuretic chlorthalidone has been shown to be superior to ACE-I and CCB in decreasing left ventricle mass and preventing heart failure in hypertensive patients. Also, we put forth the paradoxical finding that mere regression of LVH may not always translate into reduction in risk of HF; and that the pleiotropic effects of chlorthalidone may be the explanation behind its beneficial action in HF.

Dear Editor

We have read with interest the Korean Society of Hypertension guidelines for the management of hypertension: part II-diagnosis and treatment of hypertension by Lee HY, et al. [1] and congratulate the Society for an extensive review of literature while drafting the guidelines. We wish to stress upon the importance of HTN control as stated in the Guidelines - ‘The purpose of HTN treatment is to prevent CVD caused by increased BP and to reduce mortality by controlling high BP’. However, we would like to put forth following comments on clinically-crucial aspects of hypertension management:

1. It is well established that chronically increased LV workload in hypertensive patients triggers cardiac remodelling, development of LVH, increased risk of heart failure with preserved ejection fraction (HFpEF) and heart failure with reduced ejection fraction (HFrEF) and, ultimately, death [2, 3]. Thus, HT induces a compensatory thickening of the ventricular wall to normalize wall stress, which results in LV concentric hypertrophy, leading to decrease in the LV compliance and LV diastolic filling. This diastolic dysfunction has been recognised as a component of diastolic heart failure and a critical link between hypertension and heart failure [4]. Till date, there is no successful therapy for diastolic heart failure and strategies directed towards prevention of this progression from hypertension to LVH and subsequent HFpEF hold the greatest promise for reducing the burden of HF.
2. Table 11 in the Guidelines [1] titled ‘Compelling indications for choosing the antihypertensive drugs’ describes appropriate drugs according to the patient’s combined risk factors and co-morbidities. In this table, left ventricular hypertrophy (LVH) has been denoted as a compelling indication for ACE-I/ARBs and calcium channel blockers (CCBs). Surprisingly, diuretics have not been marked indicating a preference of these agents over diuretics in patients with LVH. However, landmark NIH-sponsored hypertension trials have clearly demonstrated superiority of the thiazide-like diuretic chlorthalidone (CTD) over ACE-I and CCB in reduction of left ventricle mass (LVM) and prevention of HF.
  The Treatment of Mild Hypertension Study (TOMHS) assessed the effect of five antihypertensive monotherapies (CTD, acebutolol, doxazosin, amlodipine and enalpril) on reduction of LVM in 902 patients with “mild” (stage 1) hypertension [5]. After 4 years of treatment, all 5 therapies showed reduction in LVM from baseline; but only CTD declined LVM significantly more than placebo. Average decreases ranged from 34 g for participants given CTD to 23 g for participants given enalapril and 25 g with amlodipine (*P* = 0.05 for difference among the five drug-treatment groups). It was also observed that CTD caused a significantly larger decrease in LV internal dimension at end diastole compared to other drug treatments (*P* = 0.02) including amlodipine.
  ALLHAT (Antihypertensive and Lipid-Lowering Treatment to Prevent Heart Attack Trial), the largest randomized hypertension outcomes trial (*n* = 42,418), provides another head-to-head comparison between CTD, ACE-I lisinopril and the CCB amlodipine [6]. After a mean follow-up of almost 5 years, although there was no difference between treatments on the primary outcome (combined fatal CHD or nonfatal myocardial infarction), the most intriguing finding of ALLHAT has been the significantly lower rates of HF events in the CTD group compared to both CCB and ACE-I. The amlodipine group had 38% higher risk of HF (*P* < 0.001) and 35% higher risk of hospitalized/fatal HF (*P* < 0.001) as compared to CTD. The lisinopril group had 19% higher risk of HF (*P* < 0.001) and 10% higher risk of hospitalized/fatal HF (*P* = 0.11) as compared to the CTD group. These results held true when examined across the predefined subgroups of age, race, sex, diabetes status and by absence or presence of CHD at baseline. Remarkably, the Kaplan–Meier event curves for patients hospitalized for HF or death due to HF begin to separate immediately after randomization, with relative risk of 2.22 (95% confidence interval [CI], 1.69–2.91; *P* < .001) for amlodipine vs. CTD in the first year and of 2.08 (95% CI, 1.58–2.74; *P* < .001) for lisinopril vs. CTD [7].
  Apart from TOMHS and ALLHAT, treatment with CTD has been associated with favourable changes in LVH in several other landmark hypertension trials, including the Hypertension Detection and Follow-Up Program (HDFP), the Systolic Hypertension in the Elderly Program (SHEP), and the Multiple Risk Factor Intervention Trial (MRFIT) [8–10].
3. The LVH-HF Paradox:
  The direct cardiac effect of LVH includes an increased risk of developing congestive heart failure and the associated morbidity and mortality [11]. Thus, one of the major objectives in achieving regression of LVH and reduction of LV mass is to reduce the incidence of HFpEF and HFrEF and the allied hospitalisations and death. However, reduction in LVH may not always translate into direct reduction in incidence of diastolic dysfunction and HF. The examples of this are perhaps amlodipine and the RAAS inhibitors. In the ASCOT-BPLA (Anglo-Scandinavian Cardiac Outcomes Trial-Blood Pressure Lowering Arm) study, amlodipine ± perindopril arm vs. atenolol ± bendroflumethiazide resulted in significant reductions in several secondary endpoints except fatal and non-fatal heart failure [12]. In a recent sub-study of ASCOT, it was seen that, despite regression of LVH, there was no associated improvement in diastolic function with amlodipine ± perindopril-based therapy [13]. Similarly, while inhibition of the RAAS has been shown to be beneficial in the treatment of patients with systolic HF, RAAS blockade in the prevention and treatment of HFpEF has been disappointing. In a sub-analysis of the ALLHAT, the thiazide-like diuretic CTD was shown to significantly reduce the risk of HFpEF by 31 and 26% compared to amlodipine and lisinopril, respectively [corresponding HRs and 95% CIs were 0.69 (0.53–0.91; *p* = 0.009) and 0.74 (0.56–0.97; *p* = 0.032)] [14]. Further, perindopril in PEP-CHF (Perindopril in Elderly People with Chronic Heart Failure), candesartan in CHARM-Preserved (Candesartan in Heart failure: Assessment of Reduction in Mortality and morbidity) and irbesartan in I-PRESERVE (Irbesartan in Heart Failure with Preserved Ejection Fraction Study) have all failed to show any significant difference vs. placebo in reducing the primary outcome of death and HF-related hospitalization in patients with HFpEF [15–17].
4. An interesting recent analysis of the ALLHAT data-set looked at the extent to which the effect of an anti-hypertensive on incident HF is mediated by evolving LVH. The researchers found that the beneficial effects of CTD in reduction of HF were entirely independent of evolving LVH, leading them to postulate that that the mechanisms by which chlorthalidone prevented HF were not restricted to prevention of LVH [3]. In contrast to other anti-hypertensives and diuretics, CTD has unique pleiotropic properties including improving endothelial function, reducing inflammatory and oxidative stress, inhibition of platelet aggregation and vascular permeability, and promotion of angiogenesis [18]. These features probably impart CTD a distinctive advantage to not only effectively reduce the BP and LVH, but also prevent progression to HFpEF and ultimately HF.
5. Other hypertension guidelines: Finally, it is important to discuss similar recommendations from other recent hypertension guidelines.
  - The ACC-AHA 2017 Guidelines [19] state that “LVH is a secondary manifestation of hypertension and independently predicts future CVD events. BP lowering leads to a reduction in LV mass. In TOMHS (Treatment of Mild Hypertension Study), the long-acting diuretic chlorthalidone was slightly more effective in reducing LVH than were a calcium channel blocker (CCB) (amlodipine), ACE inhibitor (enalapril), alpha-receptor blocker (doxazosin), or beta-receptor blocker (acebutolol). Beta blockers are inferior to angiotensin receptor blockers (ARBs), angiotensin converting enzyme (ACE) inhibitors, and CCBs in reducing LVH.”
  - The ESC-ESH 2018 Guidelines while discussing left ventricular hypertrophy and heart failure observe that “Treating hypertension has a major impact on reducing the risk of incident heart failure and heart failure hospitalization, especially in old and very old patients. This has been observed using diuretics, beta-blockers, ACE inhibitors, or ARBs, with CCBs being less effective in comparative trials. Reducing BP can also lead to the regression of LVH, which has been shown to be accompanied by a reduction of CV events and mortality. The magnitude of LVH regression is associated with baseline LV mass, duration of therapy, the SBP reduction, and the drugs used, with ARBs, ACE inhibitors, and CBBs causing more effective LVH regression than beta-blockers or diuretics” [20]. However, the guideline authors do not provide any reference for stating RAS blockers or CCBs as superior over diuretics. They do cite a reference for superiority over beta-blockers.
  - Canada Hypertension Guidelines 2018 recommend that in treatment of HT in association with LVH “Initial therapy can be drug treatment using ACE inhibitors, ARBs, long-acting CCBs, or thiazide/thiazide-like diuretics. Antihypertensive treatment should include an agent from one of the five major classes in patients with LVH, with care given to the inferiority of β-blockers in patients with LVH” [21].
  - The 2016 National Heart Foundation of Australia Guidelines [22] state that “In a meta-analysis of differences between antihypertensive treatments, heart failure was more likely to occur in those given calcium channel blockers compared to those given diuretics, ACE inhibitors or beta-blockers. A network meta-analysis involving 223,313 patients published in 2011 also reported diuretics as the most effective class of drugs in preventing heart failure”.

To summarize, we wish to highlight that data from head-to-head landmark studies suggests that CTD has superior effects than ACE-I and CCBs in reduction of LV mass and risk of HF amongst hypertensive patients. We have raised similar concerns about CCBs in diabetic hypertensive patients with diastolic dysfunction - thiazide-like diuretics should be preferred ahead of CCBs for blood pressure control in such patients to prevent HF [23]. Similarly, in view of the discussed evidence, we urge the Korean Society of Hypertension to relook at and suitably amend their recommendations for hypertensive patients with LVH.

## Data Availability

Not applicable.

## Abbreviations

ACC-AHA: American College of Cardiology and American Heart Association
ACE-I: Angiotensin converting enzyme inhibitors
ALLHAT: Antihypertensive and Lipid-Lowering Treatment to Prevent Heart Attack Trial
ARB: Angiotensin Receptor Blocker
CCB: Calcium Channel Blocker
CTD: Chlorthalidone
ESC-ESH: European Society of Cardiology and European Society of Hypertension
HF: Heart failure
HFpEF: Heart failure with preserved ejection fraction
HFrEF: Heart failure with reduced ejection fraction
HTN: Hypertension
LVH: Left ventricular hypertrophy
NIH: National Institutes of Health
RAAS: Renin–angiotensin–aldosterone system
TOMHS: Treatment of Mild Hypertension Study
